# Protective Role of Vitamin D in Renal Tubulopathies

**DOI:** 10.3390/metabo10030115

**Published:** 2020-03-19

**Authors:** Guido Gembillo, Valeria Cernaro, Rossella Siligato, Francesco Curreri, Antonino Catalano, Domenico Santoro

**Affiliations:** 1 Unit of Nephrology and Dialysis, Department of Clinical and Experimental Medicine, University of Messina, Via Consolare Valeria, 98125 Messina, Italy; valecern82@virgilio.it (V.C.); rossellasiligato@gmail.com (R.S.); 2 MIFT, University of Messina, 98125 Messina, Italy; fcurreri@unime.it; 3 Department of Clinical and Experimental Medicine, University of Messina, 98125 Messina, Italy; catalanoa@unime.it

**Keywords:** Vitamin D, Tubulopathies, Tubular Injury, Megalin, Cubilin, FGF23, Klotho, Calcitriol, CKD, VDR

## Abstract

Vitamin D is tightly linked with renal tubular homeostasis: the mitochondria of proximal convoluted tubule cells are the production site of 1α,25-dihydroxyvitamin D3. Patients with renal impairment or tubular injury often suffer from chronic inflammation. This alteration comes from oxidative stress, acidosis, decreased clearance of inflammatory cytokines and stimulation of inflammatory factors. The challenge is to find the right formula for each patient to correctly modulate the landscape of treatment and preserve the essential functions of the organism without perturbating its homeostasis. The complexity of the counter-regulation mechanisms and the different axis involved in the Vitamin D equilibrium pose a major issue on Vitamin D as a potential effective anti-inflammatory drug. The therapeutic use of this compound should be able to inhibit the development of inflammation without interfering with normal homeostasis. Megalin-Cubilin-Amnionless and the FGF23-Klotho axis represent two Vitamin D-linked mechanisms that could modulate and ameliorate the damage response at the renal tubular level, balancing Vitamin D therapy with an effect potent enough to contrast the inflammatory cascades, but which avoids potential severe side effects.

## 1. Introduction

Vitamin D is a fundamental agent for body homeostasis and maintaining a proper level of this hormone could make the difference in strategies against many systemic diseases; however, the best way to reach the proper level of this hormone is still debated. 

The USA Endocrine Society recommends a dietary intake of 600 IU/day for adults in the general population and to double or triple the dose in patients at risk of Vitamin D deficiency; they define as adequate a level of 75–250 nmol/L (30–100 ng/mL) of Vitamin D [1]. The Institute of Medicine (IOM) identified as a proper level of Vitamin D a lower threshold of ≥50 nmol/L ( ≥ 20 ng/mL) [2]. In line with IOM findings, the National Osteoporosis Society recommends to reach a level >50 nmol/L of Vitamin D to obtain an adequate status [3].

The National Kidney Foundation in the Kidney Disease Outcomes Quality Initiative (K/DOQI) clinical practice guidelines for bone metabolism and disease in chronic kidney disease (CKD) encourages maintaining a value of Vitamin D above 30 ng/mL. The K/DOQI also suggests that for desirable serum 25(OH)D levels, supplementation with Vitamin D should be initiated using dosing regimens recommended for the general population [4].

Correct homeostasis of the renal tubular function is fundamental to guarantee the right balance of intake, production and excretion of Vitamin D. Renal tubules have a central role in the modulation of Vitamin D activity for different reasons: Vitamin D binding protein (DBP) endocytosis is linked to the interaction of Megalin, Cubilin and Amnionless complex, three key regulators of proximal renal tubules expressed in the apical membrane [5].

On the basolateral membrane of renal tubular cells, Fibroblast Growth Factor 23 (FGF23) triggers the signaling cascade that inhibits the 1α-hydroxylase activity, which is the fundamental enzyme that operates the activation of Vitamin D. FGF23 exerts its action binding to the complex of FGF receptors (FGFRs) and αKlotho hormone. Different pathologic entities that affect renal tubules could be linked to Vitamin D dysregulation. Reaching homeostasis in Vitamin D balance could be a pivotal strategy in the treatment of these diseases [6]. 

### 1.1. Vitamin D: A Polymorphous Agent with Heterogeneous Functions

Vitamin D and, in particular, its active form 1,25-dihydroxyvitamin D [1,25(OH)2D3], is a fundamental regulator of Calcium and Phosphate balance: it controls Calcium levels influencing apical intestinal Calcium and distal convoluted tubule uptake via transient receptor potential vanilloid modulation [7], 1,25(OH)2D3 also promotes phosphorus absorption from intestinal lumen through both non-active and sodium-dependent transport pathways. At the same time, 1,25(OH)2D3 regulates plasma parathyroid hormone (PTH) levels that, in turn, contribute to modulate Calcium and Phosphate metabolism [8]. 1,25(OH)2D3 exerts negative feedback on PTH decreasing its production and release, while, *au contraire*, PTH secretion increases 1,25(OH)2D3 production [9,10].

Vitamin D exerts numerous pleiotropic effects unrelated to plasma calcium homeostasis. Indeed, various studies demonstrated that this hormone and its active analogs perform relevant anti-proteinuric, anti-fibrotic and anti-inflammatory actions in subjects with CKD and in experimental studies on animals with end-stage renal disease [11,12,13].

The active form of Vitamin D is produced by mitochondria of the renal proximal convoluted tubules. In tubular epithelial cells, which have a preeminent function in the synthesis of active Vitamin D [14], 1α-hydroxylase converts 25-hydroxyvitamin D [25(OH)D] to [1,25 (OH)2D3] [15].

Most of the Vitamin D moves in the circulation by the Vitamin D-binding protein (DBP), in a smaller percentage it is carried by blood proteins—albumin and lipoproteins—and in a minimum part, it flows freely in its biologically active form. These complexes binding the Vitamin D are filtered through the glomeruli, after which megalin can re-uptake it in the proximal tubule; for this reason, an acute or chronic tubular injury could be associated to a loss of Vitamin D [16].

Vitamin D has many pleiotropic effects on body homeostasis, such as cardio-protective and neuro-protective functions, hypertension control and is also involved in antitumor activity as well as immunomodulation [17,18,19] through the Vitamin D receptor (VDR). VDR has a fundamental part in the kidney homeostasis, since its activation protects against renal injury in kidney diseases regulating the renin–angiotensin–aldosterone system (RAAS) activation and anti-inflammatory pathways, inhibiting renal fibrogenesis, maintaining podocyte health, restoring mitochondrial function and suppressing autoimmunity and renal cell apoptosis [20,21,22].

Several case reports underlined the link between renal tubular acidosis and deficit of Vitamin D, this condition leads to inappropriate reabsorption of bicarbonate and perturbance of electrolyte pool homeostasis [23,24,25].

A proper level of Vitamin D has a protective role on the proximal renal tubule; this status could contribute to the balance of blood pH: in fact, in this segment of nephron, bicarbonate buffer is reabsorbed through a mechanism coupled to the secretion of hydrogen into the lumen [26]. 

### 1.2. Vitamin D Protective Role in Tubular Damage in CKD

Vitamin D plays a pivotal role at a tubular level, and VDRs are present in both proximal and distal nephron [27].

It modulates the 1a-hydroxylase and 24-hydroxylase enzymes in the proximal tubule [28,29] and stimulates calbindin-D28K and Ca^2+^ reabsorption in the distal tubule [30,31].

A challenging question about Vitamin D action is how it can influence the tubular damage and renal fibrosis typical of CKD [32]. 

CKD is characterized by myofibroblast generation and activation. These mechanisms are influenced by several inflammatory mediators such as the cytokine transforming growth factor β (TGFβ), a powerful inducer of myofibroblast differentiation [33]; myofibroblast formation could also derive from epithelial and endothelial cells through the epithelial/endothelial-mesenchymal (EMT/EndMT) transition, with consequences such as renal fibrogenesis and tubular atrophy, secondary to a decreased epithelial cell mass and an increase in the matrix-producing effector cells [34]. 

Myofibroblast activation could be triggered by paracrine and autocrine mechanisms: lymphocyte and macrophage signals can stimulate their activation as well as autocrine factors generated even by myofibroblasts and pathogen-associated molecular patterns derived from pathogenic agents that cooperate through pattern recognition receptors on fibroblasts. 

Cytokines, chemokines, angiogenic factors, acute phase proteins, caspases and components of the renin–angiotensin–aldosterone system (RAAS) have been established as significant mediators of fibrotic damage [35].

Interstitial fibrosis also impairs the ability of tubules to secrete toxins from peritubular capillaries, interferes with their passage to the capillaries and impairs the exchange of nutrients from the circulatory system [36].

In an experimental mouse model, Arfian et al. [37] observed that treatment with 1,25-dihydroxyvitamin D was significantly associated with lower fibrotic status, tubular impairment scores and myofibroblast proliferation compared with the control group. 

Vitamin D deficiency intensifies tubulointerstitial injury and degeneration in interstitial fibrosis after ischemia/reperfusion insult linked to an elevation of PTH, a decrease of plasma FGF-23, VDR receptor and Klotho protein and to an increased expression of pro-inflammatory factors such as transforming growth factor β (TGF-β) [38]. Inoue et al. [39] confirmed these results in their study, showing how Vitamin D analog 22-oxacalcitriol has a protective role from tubulointerstitial fibrosis by containing the autoinduction of TGF-β1. 

Moreover, Lee at al [40] outlined how paricalcitol attenuates functional deterioration and histological damage during induced AKI in mice. Vitamin D administration considerably lowed tissue neutrophil and macrophage infiltration and chemokines levels.

Proximal tubule epithelial cells (PTECs) are the most expressed cell type in the kidney and are responsible for the reabsorption of 60%–70% of water and NaCl, 80% of NaHCO3 and almost nutrients present in the glomerular ultrafiltrate, fluid-electrolyte and acid-base balance, ammoniagenesis, Vitamin D synthesis and immunomodulating functions [41]. Injured PTECs and epithelial-to-mesenchymal transformation of PTECs induce pro-inflammatory and inflammatory cytokines production [42].

Active Vitamin D is able to up-regulate the protein kinase B/mitochondrial uncoupling protein 2 (AKT/UCP2) signaling, which reduces oxidative stress of renal tubular epithelial cell line (HK2) [43]. UCP2 is a protein, mainly located in proximal convoluted tubule cells, that has been demonstrated to have a pivotal role in the preservation of mitochondria, immune regulation and modulation of oxidative stress under physiological or pathological conditions affecting the kidney [44,45]. 1,25-dihydroxyvitamin D exerts a direct protective effect, significantly decreasing IL-6 and IL-8 levels in PTECs and modulating the activity of effector T-cells [46]. 

Calcitriol has been demonstrated to be strongly associated with the inhibition of NF-κB and necroptotic pathway, via upregulating A20 in Ang II-induced renal injury [47]. Zinc finger protein A20 is a strong NF-κB pathway negative regulator and necroptosis inhibitor [48]; A20 induction primarily occurs in the tubular epithelial cells and its proper control leads to manage inflammation and ROS generation. In rat PTECs, 1,25-dihydroxyvitamin D tightly upregulated A20 and reduced pro-inflammatory cytokines and ROS.

Zhang et al. [49] demonstrated in vitro that 1,25-dihydroxyvitamin D and its analog Elocalcitol (BXL-628) have a relevant role in the modulation of RhoA/Rho (member of the Rho guanosine triphosphatases family), associated protein kinase pathway (ROCK) in human renal proximal tubular cells. Zhang et al. also found out in a previous study [50] involving HK-2 cells in vitro that the TGF-β1-mediated RhoA/ROCK activation contributed to the dissolution of tight junctions during EMT, which is one of the mechanisms underlying renal fibrosis [51].

1,25-dihydroxyvitamin D also regulates the production of IL-8 and MCP-1 by human primary PTECs [52]; these chemokines exert a relevant action on tubular damage as demonstrated in several renal injuries [53,54,55], underlining their contribute to inflammatory processes in the tubulointerstitium. Chung et al. [46] proved that 1,25(OH)2D3 may have a protective function on effector CD4+ T cells or inflammatory cytokine-induced injury in human renal PTECs. Indeed, treatment with 1,25(OH)2D3 significantly reduced TNF-α, IL-17 and TGF-β levels (*p* < 0.05 for all). 

Paricalcitol administration attenuates 4-hydroxy-2-hexenal-induced renal tubular cell damage by suppressing inflammation and EMT process through inhibition of the NF-κB (nuclear factor-κB), TGF-β/Smad and β-catenin signaling pathways in human renal PTECs [56].

Activation of VDR by paricalcitol protects against lipopolysaccharide (LPS)-induced acute kidney injury by blocking renal tubular epithelial cell apoptosis and preventing LPS-stimulated caspase 3 activation in the renal cortex of LPS-treated mice [57]. Paricalcitol administration also contributes to the modulation of renal inflammatory infiltration and RANTES (Regulated on Activation, Normal T Cell Expressed and Secreted) expression by promoting VDR-mediated sequestration of NF-κB signaling [58].

AKT, a reactive oxygen species (ROS) target, has a pivotal role in controlling cell growth and proliferation, and its downregulation contributes to renal proximal tubular cell apoptosis [59]: Hong et al. [60] established the important function of paricalcitol through the anti-inflammatory and antiapoptotic effects of AKT and NF-κB signaling in LPS-induced renal proximal tubule cell injury. 

In their study, Morgado-Pascual et al. [61] found out that paricalcitol could also exert an anti-inflammatory action in cultured tubular epithelial cells through the inhibition of “a disintegrin and metalloproteinase” (ADAM17)/epidermal growth factor receptor (EGFR) signaling axis. Their group demonstrated in an experimental model [62] that this ADAM17/EGFR pathway contributes to renal inflammation and the modulation of its overexpression could lead both to better regulation of fibrosis and to the inhibition of aldosterone-mediated proinflammatory factors overexpression.

## 2. Megalin, Cubilin and Vitamin D: A Synergy into Proximal Tubule Cells for Body Homeostasis 

Megalin, also known as Lrp2 (low-density lipoprotein-related protein 2) or gp330, is a transmembrane receptor for serum DBP in renal cells and is essential for the uptake of the 25(OH)D3-DBP complex [63]. The 58-kD DBP is the main transporter for Vitamin D_3_ metabolites in the circulation. DBP presents the highest affinity for 25-(OH) Vitamin D_3_ (*K*_d_ = 10^−10^ to 10^−12^ M). Due to this tight binding affinity and the high plasma concentration of DBP (0.3 to 0.5 mg/mL), almost all circulating 25-(OH) Vitamin D_3_ molecules are present in complex with DBP [64].

Megalin also mediates the cellular uptake of many serum transport proteins, including those that act as carriers for the steroid hormone androgen and estrogen as well as lipophilic vitamins such as retinol [65,66].

Studies in rodent models showed that the DBP-Vitamin D complex is constantly filtered through the glomerulus and endocytosed via megalin into PTECs, leading to the activation of 25(OH)D(3) to the active form [67]. Chapron et al. [68], using a human cell-derived model, confirmed that 25OHD3 is transported into the human proximal tubule epithelium via megalin-mediated endocytosis while bound to DBP. 

Loss of this receptor in congenital or acquired diseases results in multiple organ dysfunctions, including forebrain malformation (holoprosencephaly) and the Donnai-Barrow/Facio-Oculo-Acustico-Renal syndrome characterized by a multifaceted phenotype including low-molecular-weight proteinuria [69] and renal reabsorption defects (renal Fanconi syndrome) [70]. Beydoun et al. [71] also demonstrated that VDR and megalin gene variations can modify age-related cognitive decline among US adults. De et al. [72] in their study underlined how urinary full length megalin (C-megalin) excretion is increased via exocytosis from renal PTECs injured by endo-lysosomal overload caused by megalin-mediated protein metabolism.

Toi et al. [73] studied C-megalin excretion in 153 pre-dialysis CKD patients finding that its determination is clinically relevant for the assessment of renal PTECs injury. This hypothesis was based on the assessment of the correlation between C-megalin urinary excretion, excretion of β2 microglobulin/Creatinine ratio and Serum α1 microglobulin/Creatinine ratio all markers for renal PTECs dysfunction; they also enlightened that urinary loss of C-megalin might be linked to the reduction of serum 25(OH)D.

**Figure 1:**
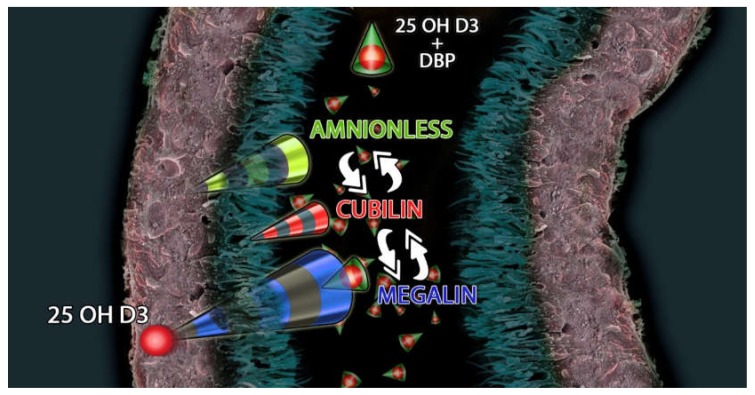
Megalin-Cubilin-Amnionless interacts with 25(OH)D3 and DBP (Vitamin D Binding Protein) complex to modulate the uptake of 25(OH)D3 in the proximal tubule.

Megalin cooperates with various intracellular proteins and other membrane molecules, including the cubilin-amnionless complex [74] (Figure 1). 

Cubilin is a structurally unique, peripheral membrane protein [75] co-expressed with megalin in the apical endocytic compartments of the proximal tubule [76]. It is another endocytic DBP receptor and its genetic defects may induce urinary loss of DBP and a decrease in plasma Vitamin D levels [77,78].

Bonnet et al. [79] reported, in an experiment on rodents, that cubilin expression is mediated by a VDR-dependent mechanism and that cubilin has a major role in 25(OH)D3 uptake by adipocytes. In these cells, Vitamin D not only is stored in lipid droplets but could also be converted to active metabolites modulating adipocyte biology and 25(OH)D3 mobility [80]. 

Dogs with an inherited disorder affecting cubilin biosynthesis exhibit abnormal Vitamin D metabolism; in the same way, human patients with mutations causing cubilin dysfunction show abnormal urinary excretion of 25(OH)D3 [78]. 

Amsellem et al. [81], by employing a set of genetic models allowing inactivation of cubilin, megalin or both simultaneously, described the essential role of cubilin in albumin reabsorption by proximal tubule cells and described the mechanism of internalization of cubilin-albumin complexes. This function of cubilin has a relevant role in Vitamin D homeostasis; indeed, only 85% of Vitamin D is bound to DBP, there is a 15% bound to albumin [82]. 

## 3. FGF23-Klotho and Vitamin D Axis in Endocrine-Paracrine Renal Loop 

FGF23, Vitamin D and Klotho have a central role in the homeostasis of tubular function, primarily regulating renal calcium and phosphate reabsorption. When the phosphate level is increased in the urine, this will contribute to tubular injury and interstitial fibrosis [83].

FGF23 is a hormone mainly produced and secreted by osteocytes and osteoblasts, even if different tissues can generate it [84,85]. FGF23 is crucial in the phosphorus homeostasis and acts through interactions with the FGF23 co-receptor α-Klotho (KL) and FGF receptors in the kidney [86]. FGF23 is a primary α-klotho-dependent endocrine regulator of mineral homeostasis, functioning to activate Vitamin D and as a phosphatonin [87]. 

Genetic inactivation of FGF23, klotho or both of the genes has resulted in markedly increased kidney expression of 1-alpha hydroxylase and concomitant elevated serum levels of 1,25(OH)2D3 [88].

In the early stages of CKD, serum FGF23 levels can rise 1000-fold above normal values. This condition is determined by the need to maintain acceptable phosphate levels [89]. The overexpression of FGF23 is linked to hypophosphatemic rickets/osteomalacia, in contrast, a decrease in the action of FGF23 results in hyperphosphatemic calcinosis with high 1,25(OH)2D3 levels [90]. Vitamin D, through its action on VDR, regulates the promoter region in the FGF23 gene and locally produced 1,25(OH)2D3 in bone cells regulates FGF23 generation [91,92]. 25(OH)D3 hydroxylation is inhibited by FGF23 through the action of extracellular signal-regulated kinase 1/2 (ERK 1/2) [93].

FGF23 performs a phosphaturic action through the inhibition of phosphate proximal tubular resorption by sodium phosphate cotransporters NaPi2a and NaPi2b [94]. This phosphate reabsorption inhibition is mediated by a Klotho-dependent activation of extracellular signal-regulated ERK1/2 and of serum/glucocorticoid-regulated kinase-1, leading to phosphorylation of the scaffolding protein Na^+^/H^+^ exchange regulatory cofactor and internalization/degradation of sodium-phosphate cotransporters [95].

**Figure 2:**
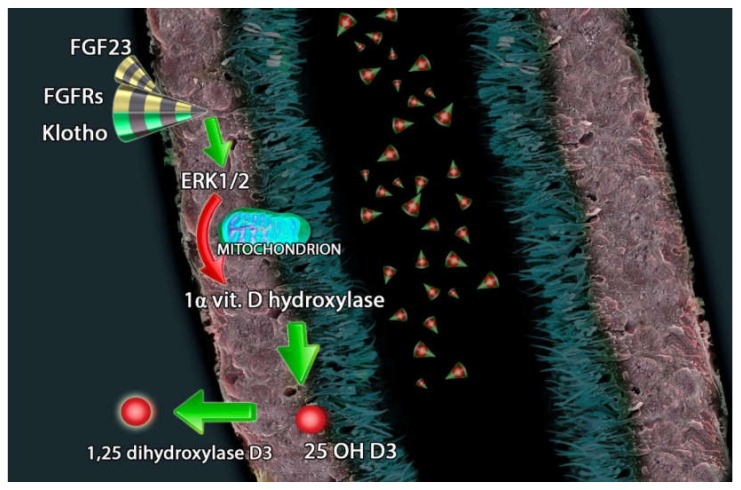
FGF23 (fibroblast growth factor-23), FGFRs (FGF receptors) and Klotho counter regulate the hydroxylation of 25(OH)D3 through extracellular signal-regulated kinases 1/2 and the inhibition of 1-alpha-hydroxylase.

Furthermore, FGF23 reduces renal production of 1,25(OH)2D3 [85] and PTH secretion [96]. FGF23 suppresses the expression of 1α-hydroxylase enzyme in the kidney and induces the synthesis of 25-hydroxyvitamin D24-hydroxylase enzyme; this mechanism leads to a reduced conversion of 25(OH)D into 1,25(OH)2D and increased catabolism of 25(OH)D and 1,25(OH)2D3 into inactive carboxylic acids [97,98] (Figure 2).

The Paracalcitol and ENdothelial fuNction in chronic kidneY disease (PENNY) study [99] showed that paricalcitol reduced serum PTH (−75.1 pg/mL, 95% CI −90.4 to −59.8; *p* < 0.001) and its action is linked to increased FGF23 levels ( + 107 pg/mL, 95% CI 44 to 170; *p* = 0.001). Changes in the Ca × P product in response to paricalcitol were also closely related to concurrent FGF23 (*p* < 0.001).

Charoenngam et al. [100] in their meta-analysis also demonstrated that serum intact FGF23 concentration increased strongly after oral 1,25(OH)2D3 supplementation in Vitamin D-deficient participants, with a pooled standardized mean difference of 0.36 (95%CI 0.14 to 0.57; *p* = 0.001). 

FGF23 works in close synergy with α-Klotho, a multifunctional protein that is predominantly expressed in kidney tubular epithelium and is also implicated in the metabolism of phosphate, calcium and Vitamin D. In the study of Ozeki et al. [101] on a cohort of cardiac patients with CKD, estimated glomerular filtration rate (eGFR) was correlated negatively with FGF23 and positively with α-Klotho serum levels.

There is also evidence that Klotho may regulate renal phosphate reabsorption by an FGF23 independent mechanism [102]. Hu et al. [103] reported that Klotho can act as an autocrine phosphaturic factor by altering the function of NaPi2a in renal PTECs through its glucuronidase activity.

Qian et al. [104] demonstrated that recombinant Klotho treatment protects renal tubular epithelial cells from ischemic-reperfusion injury by inhibiting the oxidative stress that can provoke necroptotic cell death in acute kidney injury. 

Immunoreactivity of Klotho is closely associated with proliferation in the intercalated cells of the connecting tubules and cortical collecting ducts and may be implicated in the regulation of tubular proliferation [105].

The close link between Vitamin D and the FGF23/Klotho axis needs further investigation to fully understand the optimal strategy to prevent, retard and inhibit the comorbidity of tubular and renal dysfunction. 

## 4. Vitamin D in Primitive Renal Tubular Disorders

Primitive renal tubular disorders are a group of diseases leading to fluid loss and abnormalities in the electrolyte and acid-base balance.

Many tubulopathies cause chronic dehydration, salt wasting or acidosis, while excessive phosphate loss leads to rickets in young patients and altered bone development [106].

One of the most investigated tubulopathies is Fanconi syndrome. This is characterized by a massive dysfunction of the proximal tubule that leads to glucosuria, phosphaturia, generalized aminoaciduria, and type 2 renal tubular acidosis [107]. Primary inherited Fanconi syndrome is provoked by a mutation in the NaP_i_-II in the proximal tubule. Recent studies have established new causes of Fanconi syndrome due to mutations in the *EHHADH* and the *HNF4A* genes [108].

Taylor et al. [109] reported the case of an adult patient showing type 2 renal tubular acidosis with Fanconi syndrome, osteomalacia, osteoporosis and secondary hyperaldosteronism. After 24 months of Cholecalciferol and calcium therapy, the lumbar spine T-score improved to −2.0, and the femoral neck T-score improved to −2.7. Bone biopsy specimens demonstrated the resolution of the osteomalacia. Ali et al. [110] also presented the case of a young female who had type 2 renal tubular acidosis and Fanconi syndrome and excellently responded to Cholecalciferol treatment. Baran et al. [111] described a case of incomplete Fanconi syndrome (without metabolic acidosis) treated with 1,25(OH)2D3 and hydrochlorothiazide with improvement in calcium and phosphorus balance. 

Another inherited renal tubular disorder is Bartter syndrome, which is generated by defective salt reabsorption in the thick ascending limb of the loop of Henle, leading to salt wasting, hypokalemia and metabolic alkalosis [112]. Li et al. [113] described a case of type 2 Bartter syndrome positively treated with celecoxib and Vitamin D. Krishnamurthy et al. [114] presented a case of Bartter syndrome with Vitamin D-resistant rickets, probably secondary to the calcipenic effect of hyperprostaglandinemia or phosphate loss in urine. 

In the literature, there are even two cases of Imerslund-Gräsbeck syndrome related to cubilin and amnionless (CUBAM) receptor proteins, both expressed in the small intestine as well as in the proximal tubules of the kidney. In these two cases, cubilin impairment led to loss of Vitamin D-binding protein and severe Vitamin D deficiency [115,116]. 

Vitamin D and phosphates are the conventional therapy for X-linked hypophosphatemia (XLH), an FGF23-mediated disorder characterized by impaired renal tubular reabsorption of phosphate. It is a rare affection caused by mutations in the PHEX gene. It encodes for an endopeptidase that is primarily expressed on the surface of osteoblasts, osteocytes, odontoblasts and cementoblasts [117]. XLH follows an X-linked mode of transmission with dominant expression and represents the first cause of hypophosphatemic rickets [118].

Liu et al. [95] treated mice suffering from XLH with daily 1,25(OH)2D3 repletion, FGF23 antibodies or biweekly high-dose of 1,25(OH)2D3 from d2 to d75 without supplemental phosphate to establish if 1,25(OH)2D3 or blocking FGF23 activity could improve the skeletal phenotype without phosphate supplementation. Their group demonstrated that daily 1,25(OH)2D3 supplementation was more efficient than other supplementations in regulating the expression of the growth plate and metaphyseal organization and had a relevant role in normalizing bone geometry and lumbar vertebral height and body weight.

The recent development and approval of an anti-FGF23 antibody, burosumab, for use in XLH provides a novel treatment option, alternative to Vitamin D therapy [119].

There is still much to be done for the treatment of tubulopathies, and atypical cases will continue to pose major challenges for physicians and researchers. 

## 5. Conclusions

Chronic inflammation is a common feature in patients with renal impairment or tubular injury. This alteration originates from oxidative stress, acidosis, decreased clearance of inflammatory cytokines and stimulation of inflammatory factors. The challenge is to find the right formula for each patient to correctly modulate the landscape of treatment and preserve the essential functions of the organism without perturbing its homeostasis. 

Experimental models in vitro, as well as animal and human studies, demonstrated the protective role of Vitamin D against inflammatory injuries and how the use of the different formulations of this hormone could be a tool in broader therapeutic strategies. The complexity of the counter-regulation mechanisms and the different axis involved in the Vitamin D equilibrium pose a major issue on Vitamin D as a potential effective anti-inflammatory drug. The therapeutic use of this compound should be able to inhibit the development of inflammation without interfering with normal homeostasis. Megalin-Cubilin-Amnionless and FGF23-Klotho axes represent two Vitamin D-linked mechanisms that could modulate and ameliorate the damage response at the renal tubular level, balancing Vitamin D therapy with an effect potent enough to contrast the inflammatory cascades, but which avoids potential severe side effects [120]. 

More research is needed to confirm these findings and to better understand the different anti-inflammatory pathways involved in renal fibrogenesis and how to preserve the tubular segments, fundamental for metabolic homeostasis. The potential benefits of Vitamin D in the prevention and treatment of immune and inflammatory conditions are encouraging; the current answers that experimentation and clinical practice provide us are still not satisfactory.

## Abbreviations

[1,25 (OH)_2_D_3_]	1,25-dihydroxyvitamin D3
[25(OH)D]	25-hydroxyvitamin D
ADAM17	a disintegrin and metalloproteinase
AKT/UCP2	protein kinase B/mitochondrial uncoupling protein 2
Calcidiol	25-hydroxyvitamin D
Calcitriol	1,25-dihydroxy vitamin D
CKD	Chronic Kidney Disease
CI	Confidence Interval
DB/FOAR	Donnai-Barrow/Facio-Oculo-Acustico-Renal
DBP	Vitamin D Binding Protein
eGFR	estimated glomerular filtration rate
EMT/EndMT	epithelial/endothelial-mesenchymal
ERK 1/2	extracellular signal-regulated kinase ½
FGF23	Fibroblast Growth Factor 23
FGFRs	FGF receptors
HK2	renal tubular epithelial cell line
HRPTEpiC	human renal PTECs
IL-6	cytokine interleukin-6
LPS	Lipopolysaccharide
Lrp2	low-density lipoprotein-related protein 2
MCP-1	monocyte chemoattractant protein
MIP-1β	monocyte inflammatory protein
NaP_i_-II	sodium-phosphate cotransporter
NF-κB	nuclear factor-κB
PTH	parathyroid hormone
PPARs	peroxisome proliferator-activated receptors
PDGF	platelet-derived growth factor
PICs	pro-inflammatory cytokines
PTECs	Proximal Tubular Epithelial Cells
RAAS	renin–angiotensin–aldosterone system
RANTES	regulated on activation, normal T cell expressed and secreted
ROCK	Rho protein kinase pathway
ROS	reactive oxygen species
SAP	serum amyloid P
TGFβ	transforming growth factor β
VDR	Vitamin D receptor
VEGF	vascular endothelial growth factor
XLH	X-linked hypophosphatemia

## References

[1] Holick M.F., Binkley N.C., Bischoff-Ferrari H.A., Gordon C.M., Hanley D.A., Heaney R.P., Murad M.H., Weaver C.M. (2011). Evaluation, treatment, and prevention of vitamin D deficiency: An endocrine society clinical practice guideline. J. Clin. Endocrinol. Metab..

[2] Ross A.C., Taylor C.L., Yaktine A.L., Institute of Medicine (US) Committee to Review Dietary Reference Intakes for Vitamin D and Calcium (2011). Dietary Reference Intakes for Calcium and Vitamin D.

[3] National Osteoporosis Society (2013). Vitamin D and Bone Health: A Practical Clinical Guideline for Patient Management.

[4] Kidney Disease: Improving Global Outcomes (KDIGO) CKD-MBD Work Group (2009). KDIGO clinical practice guideline for the diagnosis, evaluation, prevention, and treatment of chronic kidney disease-mineral and bone disorder (CKD-MBD). Kidney Int. Suppl..

[5] Coudroy G., Gburek J., Kozyraki R., Madsen M., Trugnan G., Moestrup S.K., Verroust P.J., Maurice M. (2005). Contribution of cubilin and amnionless to processing and membrane targeting of cubilin-amnionless complex. J. Am. Soc. Nephrol..

[6] Erben R.G. (2018). α-Klotho’s effects on mineral homeostasis are fibroblast growth factor-23 dependent. Curr. Opin. Nephrol. Hypertens..

[7] Hoenderop J.G., Nilius B., Bindels R.J. (2005). Calcium absorption across epithelia. Physiol. Rev..

[8] Sabbagh Y., Giral H., Caldas Y., Levi M., Schiavi S.C. (2011). Intestinal phosphate transport. Adv. Chronic. Kidney Dis..

[9] Lambers T.T., Bindels R.J., Hoenderop J.G. (2006). Coordinated control of renal Ca^2+^ handling. Kidney Int..

[10] Khundmiri S.J., Murray R.D., Lederer E.P.T.H. (2016). Vitamin, D. Compr. Physiol..

[11] Li Y.C. (2010). Renoprotective effects of vitamin D analogs. Kidney Int..

[12] Gembillo G., Cernaro V., Salvo A., Siligato R., Laudani A., Buemi M., Santoro D. (2019). Role of Vitamin D Status in Diabetic Patients with Renal Disease. Medicina (Kaunas).

[13] Santoro D., Sebekova K., Teta D., De Nicola L. (2015). Extraskeletal Functions of Vitamin, D. Biomed. Res. Int..

[14] Tan X., Li Y., Liu Y. (2006). Paricalcitol attenuates renal interstitial fibrosis in obstructive nephropathy. J. Am. Soc. Nephrol..

[15] Tsuprykov O., Chen X., Hocher C.F., Skoblo R., Lianghong Yin Hocher B. (2018). Why should we measure free 25(OH) vitamin D?. J. Steroid Biochem. Mol. Biol..

[16] Jean G., Souberbielle J.C., Chazot C. (2017). Vitamin D in Chronic Kidney Disease and Dialysis Patients. Nutrients.

[17] Kochupillai N. (2008). The physiology of vitamin D: Current concepts. Indian J. Med. Res..

[18] Lucisano S., Buemi M., Passantino A., Aloisi C., Cernaro V., Santoro D. (2013). New insights on the role of vitamin D in the progression of renal damage. Kidney Blood Press Res..

[19] Yang S., Li A., Wang J., Liu J., Han Y., Zhang W., Li Y.C., Zhang H. (2018). Vitamin D Receptor: A Novel Therapeutic Target for Kidney Diseases. Curr. Med. Chem..

[20] Hu H., Xu S., Hu S., Gao Y., Shui H. (2016). Effect of 1,25(OH)(2)D(3) on transdifferentiation of rat renal tubular epithelial cells induced by high glucose. Biomed. Rep..

[21] Ahmed A., Sims R.V. (2001). Proximal renal tubular acidosis associated with osteomalacia. South. Med. J..

[22] Garcia Nieto V., Sánchez Almeida E., García García M. (1996). Renal tubular dysfunction of Vitamin D deficiency rickets. Nephron.

[23] Muldowney F.P., Freaney R., McGeeney D. (1968). Renal tubular acidosis and amino-aciduria in osteomalacia of dietary or intestinal origin. Q J. Med..

[24] Skelton L.A., Boron W.F., Zhou Y. (2010). Acid-base transport by the renal proximal tubule. J. Nephrol..

[25] Santoro D., Caccamo D., Gagliostro G., Ientile R., Benvenga S., Bellinghieri G., Savica V. (2013). Vitamin D metabolism and activity as well as genetic variants of the vitamin D receptor (VDR) in chronic kidney disease patients. J. Nephrol..

[26] Kawashima H., Kurokawa K. (1982). Localization of receptors for 1,25(OH)2D3 along the rat nephron. J. Biol. Chem..

[27] Akiba T., Endou H., Koseki C., Sakai F., Horiuchi N., Suda T. (1980). Localization of 1-a-hydroxylase activity in the mammalian kidney. Biochem. Biophys. Res. Commun..

[28] Takezawa K., Moorthy B., Mandel M.L., Garancis J.C., Ghazarian J.G. (1990). Antigenic and catalytic disparity in the distribution of cytochrome P-450 dependent 25-hydroxyvitamin D3-1-a-hydroxylase and 24-hydroxylase. Histochemistry.

[29] Bindels R.J.M., Hartog A., Timmermans J., van Os C.H. (1991). Active Ca21 transport in primary cultures of rabbit kidney CCD: Stimulation by 1,25(OH)2D3 and PTH. Am. J. Physiol. Renal Fluid Electrolyte Physiol..

[30] Bouhtiauy D.L., Lajeunesse D., Brunette M.G. (1993). Effect of vitamin D depletion on calcium transport by the luminal and basolateral membranes of the proximal and distal nephrons. Endocrinology.

[31] Siaw E.K., Walters M.R. (2002). 1,25-Dihydroxyvitamin D-stimulated calmodulin binding proteins: A sustained effect on distal tubules. Am. J. Physiol. Renal Physiol..

[32] Cernaro V., Lacquaniti A., Donato V., Fazio M.R., Buemi A., Buemi M. (2012). Fibrosis, regeneration and cancer: What is the link?. Nephrol. Dial. Transplant..

[33] Carthy J.M. (2018). TGFβ signaling and the control of myofibroblast differentiation: Implications for chronic inflammatory disorders. J. Cell Physiol..

[34] Tan X., Li Y., Liu Y. (2007). Therapeutic role and potential mechanisms of active Vitamin D in renal interstitial fibrosis. J. Steroid Biochem. Mol. Biol..

[35] Wynn T.A. (2008). Cellular and molecular mechanisms of fibrosis. J. Pathol..

[36] Duffield J.S. (2014). Cellular and molecular mechanisms in kidney fibrosis. J. Clin. Invest..

[37] Arfian N., Muflikhah K., Soeyono S.K., Sari D.C., Tranggono U., Anggorowati N., Romi M.M. (2016). Vitamin D Attenuates Kidney Fibrosis via Reducing Fibroblast Expansion, Inflammation, and Epithelial Cell Apoptosis. Kobe J. Med. Sci..

[38] Goncalves J.G., de Braganca A.C., Canale D., Shimizu MH M., Sanches T.R., Moysés RM A., Volpini R.A. (2014). Vitamin D deficiency aggravates chronic kidney disease progression after ischemic acute kidney injury. PLoS ONE.

[39] Inoue K., Matsui I., Hamano T., Fujii N., Shimomura A., Nakano C., Kusunoki Y., Takabatake Y., Hirata M., Nishiyama A. (2012). Maxacalcitol ameliorates tubulointerstitial fibrosis in obstructed kidneys by recruiting PPM1A/VDR complex to pSmad3. Lab. Invest..

[40] Lee J.W., Kim S.C., Ko Y.S., Lee H.Y., Cho E., Kim M.G., Jo S.K., Cho W.Y., Kim H.K. (2014). Renoprotective effect of paricalcitol via a modulation of the TLR4-NF-κB pathway in ischemia/reperfusion-induced acute kidney injury. Biochem. Biophys. Res. Commun..

[41] Nakhoul N., Batuman V. (2011). Role of proximal tubules in the pathogenesis of kidney disease. Contrib. Nephrol..

[42] Chesney R.W. (2016). Interactions of vitamin D and the proximal tubule. Pediatr. Nephrol..

[43] Zhu X., Wu S., Guo H. (2019). Active Vitamin D and Vitamin D Receptor Help Prevent High Glucose Induced Oxidative Stress of Renal Tubular Cells via AKT/UCP2 Signaling Pathway. Biomed. Res. Int..

[44] Ding Y., Zheng Y., Huang J., Peng W., Chen X., Kang X., Zeng Q. (2019). UCP2 ameliorates mitochondrial dysfunction, inflammation, and oxidative stress in lipopolysaccharide-induced acute kidney injury. Int. Immunopharmacol..

[45] Qin N., Cai T., Ke Q., Yuan Q., Luo J., Mao X., Jiang L., Cao H., Wen P., Zen K. (2019). UCP2-dependent improvement of mitochondrial dynamics protects against acute kidney injury. J. Pathol..

[46] Chung B.H., Kim B.M., Doh K.C., Cho M.L., Kim K.W., Yang C.W. (2017). Protective effect of 1α,25-dihydroxyvitamin D3 on effector CD4+ T cell induced injury in human renal PTECs. PLoS ONE.

[47] Zhao H., Xia Y., Gan H. (2017). Calcitriol Ameliorates AngiotensinII-Induced Renal Injury Partly via Upregulating A20. Inflammation.

[48] Jaattela M., Mouritzen H., Elling F., Bastholm L. (1996). A20 zinc finger protein inhibits TNF and IL-1 signaling. J. Immunol..

[49] Zhang W., Yi B., Zhang K., Li A., Yang S., Huang J., Liu J., Zhang H. (2017). 1,25-(OH)(2)D(3) and its analogue BXL-628 inhibit high glucose-induced activation of RhoA/ROCK pathway in HK-2 cells. Exp. Ther. Med..

[50] Zhang K., Zhang H., Xiang H., Liu J., Liu Y., Zhang X., Wang J., Tang Y. (2013). TGF-β1 induces the dissolution of tight junctions in human renal proximal tubular cells: Role of the RhoA/ROCK signaling pathway. Int J. Mol. Med..

[51] Lu Q., Chen Y.B., Yang H., Wang W.W., Li C.C., Wang L., Wang J., Du L., Yin X.X. (2019). Inactivation of TSC1 promotes epithelial-mesenchymal transition of renal tubular epithelial cells in mouse diabetic nephropathy. Acta Pharm. Sin..

[52] Krüger S., Kreft B. (2001). 1,25-dihydroxyvitamin D3 differentially regulates IL-1alpha-stimulated IL-8 and MCP-1 mRNA expression and chemokine secretion by human primary PTECs. Exp. Nephrol..

[53] Jacobson S.H., Hylander B., Wretlind B., Brauner A. (1994). Interleukin-6 and interleukin-8 in serum and urine in patients with acute pyelonephritis in relation to bacterial-virulence-associated traits and renal function. Nephron.

[54] Yung S., Ng C.Y., Au K.Y., Cheung K.F., Zhang Q., Zhang C., Yap D.Y., Chau M.K., Chan T.M. (2017). Binding of anti-dsDNA antibodies to PTECs contributes to renal tubulointerstitial inflammation. Clin. Sci. (Lond.).

[55] Wada T., Yokoyama H., Su S.B., Mukaida N., Iwano M., Dohi K., Takahashi Y., Sasaki T., Furuichi K., Segawa C. (1996). Monitoring urinary levels of monocyte chemotactic and activating factor reflects disease activity of lupus nephritis. Kidney Int..

[56] Kim C.S., Joo S.Y., Lee K.E., Choi J.S., Bae E.H., Ma S.K., Kim S.H., Lee J., Kim S.W. (2013). Paricalcitol attenuates 4-hydroxy-2-hexenal-induced inflammation and epithelial-mesenchymal transition in human renal PTECs. PLoS ONE.

[57] Du J., Jiang S., Hu Z., Tang S., Sun Y., He J., Li Z., Yi B., Wang J., Zhang H. (2019). Vitamin D receptor activation protects against lipopolysaccharide-induced acute kidney injury through suppression of tubular cell apoptosis. Am. J. Physiol. Renal Physiol..

[58] Tan X., Wen X., Liu Y. (2008). Paricalcitol inhibits renal inflammation by promoting vitamin D receptor-mediated sequestration of NF-kappaB signaling. J. Am. Soc. Nephrol..

[59] Rane M.J., Song Y., Jin S., Barati M.T., Wu R., Kausar H., Li X. (2010). Interplay between Akt and p38 MAPK pathways in the regulation of renal tubular cell apoptosis associated with diabetic nephropathy. Am. J. Physiol.-Renal Physiol..

[60] Hong Y.A., Yang K.J., Jung S.Y., Chang Y.K., Park C.W., Yang C.W., Kim S.Y., Hwang H.S. (2017). Paricalcitol attenuates lipopolysaccharide-induced inflammation and apoptosis in proximal tubular cells through the prostaglandin E(2) receptor EP4. Kidney Res. Clin. Pract..

[61] Morgado-Pascual J.L., Rayego-Mateos S., Valdivielso J.M., Ortiz A., Egido J., Ruiz-Ortega M. (2015). Paricalcitol Inhibits Aldosterone-Induced Proinflammatory Factors by Modulating Epidermal Growth Factor Receptor Pathway in Cultured Tubular Epithelial Cells. Biomed. Res. Int..

[62] Rayego-Mateos S., Morgado-Pascual J.L., Sanz A.B., Ramos A.M., Eguchi S., Batlle D., Ruiz-Ortega M. (2013). TWEAK transactivation of the epidermal growth factor receptor mediates renal inflammation. J. Pathol..

[63] Gao Y., Zhou S., Luu S., Glowacki J. (2019). Megalin mediates 25-hydroxyvitamin D(3) actions in human mesenchymal stem cells. Faseb. J..

[64] Christensen E.I., Willnow T.E. (1999). Essential role of megalin in renal proximal tubule for vitamin homeostasis. J. Am. Soc. Nephrol..

[65] Matarese V., Lodish H.F. (1993). Specific uptake of retinol-binding protein by variant F9 cell lines. J. Biol. Chem..

[66] Rowling M.J., Kemmis C.M., Taffany D.A., Welsh J. (2006). Megalin-mediated endocytosis of vitamin D binding protein correlates with 25-hydroxycholecalciferol actions in human mammary cells. J. Nutr..

[67] Saito A., Iino N., Takeda T., Gejyo F. (2007). Role of megalin, a proximal tubular endocytic receptor, in calcium and phosphate homeostasis. Ther. Apher. Dial..

[68] Chapron B.D., Chapron A., Phillips B., Okoli M.C., Shen D.D., Kelly E.J., Himmelfarb J., Thummel K.E. (2018). Reevaluating the role of megalin in renal vitamin D homeostasis using a human cell-derived microphysiological system. ALTEX.

[69] Storm T., Tranebjærg L., Frykholm C., Birn H., Verroust P.J., Nevéus T., Sundelin B., Hertz J.M., Holmström G., Ericson K. (2013). Renal phenotypic investigations of megalin-deficient patients: Novel insights into tubular proteinuria and albumin filtration. Nephrol. Dial. Transplant..

[70] Willnow T.E., Christ A. (2017). Endocytic receptor LRP2/megalin-of holoprosencephaly and renal Fanconi syndrome. Pflug. Arch..

[71] Beydoun M.A., Ding E.L., Beydoun H.A., Tanaka T., Ferrucci L., Zonderman A.B. (2012). Vitamin D receptor and megalin gene polymorphisms and their associations with longitudinal cognitive change in US adults. Am. J. Clin. Nutr..

[72] De S., Kuwahara S., Hosojima M., Ishikawa T., Kaseda R., Sarkar P., Toba K. (2017). Exocytosis-Mediated Urinary Full-Length Megalin Excretion Is Linked With the Pathogenesis of Diabetic Nephropathy. Diabetes.

[73] Toi N., Inaba M., Ishimura E., Tsugawa N., Imanishi Y., Emoto M., Hirayama Y., Nakatani S., Saito A., Yamada S. (2019). Significance of urinary C-megalin excretion in vitamin D metabolism in pre-dialysis CKD patients. Sci. Rep..

[74] De S., Kuwahara S., Saito A. (2014). The endocytic receptor megalin and its associated proteins in proximal tubule epithelial cells. Membranes (Basel).

[75] Christensen E.I., Nielsen R., Birn H. (2013). From bowel to kidneys: The role of cubilin in physiology and disease. Nephrol. Dial. Transpl..

[76] Christensen E.I., Birn H., Storm T., Weyer K., Nielsen R. (2012). Endocytic receptors in the renal proximal tubule. Physiology (Bethesda).

[77] Nykjaer A., Fyfe J.C., Kozyraki R., Leheste J.R., Jacobsen C., Nielsen M.S., Ray R. (2001). Cubilin dysfunction causes abnormal metabolism of the steroid hormone 25(OH) vitamin D(3). Proc. Natl. Acad. Sci. USA.

[78] Kaseda R., Hosojima M., Sato H., Saito A. (2011). Role of Megalin and Cubilin in the Metabolism of Vitamin D3. Ther. Apher. Dial..

[79] Bonnet L., Karkeni E., Couturier C., Astier J., Dalifard J., Defoort C., Svilar L., Martin J.C., Tourniaire F., Landrier J.F. (2018). Gene Expression Pattern in Response to Cholecalciferol Supplementation Highlights Cubilin as a Major Protein of 25(OH)D Uptake in Adipocytes and Male Mice White Adipose Tissue. Endocrinology.

[80] Landrier J.F., Marcotorchino J., Tourniaire F. (2012). Lipophilic micronutrients and adipose tissue biology. Nutrients.

[81] Amsellem S., Gburek J., Hamard G., Nielsen R., Willnow T.E., Devuyst O., Nexo E., Verroust P.J., Christensen E.I., Kozyraki R. (2010). Cubilin is essential for albumin reabsorption in the renal proximal tubule. J. Am. Soc. Nephrol..

[82] Bikle D.D., Schwartz J. (2019). Vitamin D Binding Protein, Total and Free Vitamin D Levels in Different Physiological and Pathophysiological Conditions. Front. Endocrinol. (Lausanne).

[83] Kuro O.M. (2013). A phosphate-centric paradigm for pathophysiology and therapy of chronic kidney disease. Kidney Int. Suppl..

[84] Hu M.C., Shiizaki K., Kuro-o M., Moe O.W. (2013). Fibroblast Growth Factor 23 and Klotho: Physiology and Pathophysiology of an Endocrine Network of Mineral Metabolism. Annu. Rev. Physiol..

[85] Mace M.L., Gravesen E., Hofman-Bang J., Olgaard K., Lewin E. (2015). Key role of the kidney in the regulation of fibroblast growth factor 23. Kidney Int..

[86] Noonan M.L., White K.E. (2019). FGF23 Synthesis and Activity. Curr. Mol. Biol. Rep..

[87] Tan S.J., Smith E.R., Hewitson T.D., Holt S.G., Toussaint N.D. (2014). The importance of klotho in phosphate metabolism and kidney disease. Nephrology(Carlton).

[88] Razzaque M.S. (2012). FGF23, klotho and vitamin D interactions: What have we learned from in vivo mouse genetics studies?. Adv. Exp. Med. Biol..

[89] Czaya B., Faul C. (2019). The Role of Fibroblast Growth Factor 23 in Inflammation and Anemia. Int J. Mol. Sci..

[90] Fukumoto S. (2019). FGF23 and Bone and Mineral Metabolism. Handb. Exp. Pharmacol..

[91] Rodelo-Haad C., Santamaria R., Muñoz-Castañeda J.R., Pendón-Ruiz de Mier M.V., Martin-Malo A., Rodriguez M. (2019). FGF23, Biomarker or Target?. Toxins (Basel).

[92] Nguyen-Yamamoto L., Karaplis A.C., St-Arnaud R., Goltzman D. (2017). Fibroblast Growth Factor 23 Regulation by Systemic and Local Osteoblast-Synthesized 1,25-Dihydroxyvitamin D.J. Am. Soc. Nephrol..

[93] Liu E.S., Martins J.S., Raimann A., Chae B.T., Brooks D.J., Jorgetti V., Bouxsein M.L., Demay M.B. (2016). 1,25-Dihydroxyvitamin D Alone Improves Skeletal Growth, Microarchitecture, and Strength in a Murine Model of XLH, Despite Enhanced FGF23 Expression. J. Bone Miner. Res..

[94] Liu S., Quarles L.D. (2007). How fibroblast growth factor 23 works. J. Am. Soc. Nephrol..

[95] Erben R.G., Andrukhova O. (2017). FGF23-Klotho signaling axis in the kidney. Bone.

[96] Vervloet M. (2019). Renal and extrarenal effects of fibroblast growth factor 23. Nat. Rev. Nephrol..

[97] Prié D., Friedlander G. (2010). Reciprocal control of 1,25- Dihydroxyvitamin D and FGF23 formation involving the FGF23/ Klotho system. Clin. J. Am. Soc. Nephrol..

[98] Erben R.G. (2018). Physiological actions of fibroblast growth factor23. Front. Endocrinol..

[99] D’Arrigo G., Pizzini P., Cutrupi S., Tripepi R., Tripepi G., Mallamaci F., Zoccali C. (2019). FGF23 and the PTH response to paricalcitol in chronic kidney disease. Eur. J. Clin. Invest..

[100] Charoenngam N., Rujirachun P., Holick M.F., Ungprasert P. (2019). Oral vitamin D(3) supplementation increases serum fibroblast growth factor 23 concentration in vitamin D-deficient patients: A systematic review and meta-analysis. Osteoporos Int..

[101] Ozeki M., Fujita S., Kizawa S., Morita H., Sohmiya K., Hoshiga M., Ishizaka N. (2014). Association of serum levels of FGF23 and α-Klotho with glomerular filtration rate and proteinuria among cardiac patients. BMC Nephrol..

[102] Erben R.G. (2016). Update on FGF23 and Klotho signaling. Mol. Cell Endocrinol..

[103] Hu M.C., Shi M., Zhang J., Pastor J., Nakatani T., Lanske B., Razzaque M.S., Rosenblatt K.P., Baum M.G., Kuro-o M. (2010). Klotho: A novel phosphaturic substance acting as an autocrine enzyme in the renal proximal tubule. FASEB J..

[104] Qian Y., Guo X., Che L., Guan X., Wu B., Lu R., Zhu M., Pang H., Yan Y., Ni Z. (2018). Klotho Reduces Necroptosis by Targeting Oxidative Stress Involved in Renal Ischemic-Reperfusion Injury. Cell Physiol. Biochem..

[105] Song J.H., Lee M.Y., Kim Y.J., Park S.R., Kim J., Ryu S.Y., Jung J.Y. (2014). Developmental immunolocalization of the Klotho protein in mouse kidney epithelial cells. Eur. J. Histochem..

[106] Van’T Hoff W.G. (2000). Molecular developments in renal tubulopathies. Arch. Dis. Child..

[107] Foreman J.W. (2019). Fanconi Syndrome. Pediatr. Clin. North. Am..

[108] Kashoor I., Batlle D. (2019). Proximal renal tubular acidosis with and without Fanconi syndrome. Kidney Res. Clin. Pr..

[109] Taylor H.C., Elbadawy E.H. (2006). Renal tubular acidosis type 2 with Fanconi’s syndrome, osteomalacia, osteoporosis, and secondary hyperaldosteronism in an adult consequent to vitamin D and calcium deficiency: Effect of vitamin D and calcium citrate therapy. Endocr. Pr..

[110] Ali S.A., Tariq M. (2016). Successful treatment of proximal renal tubular acidosis and Fanconi syndrome with vitamin D replacement. Saudi J. Kidney Dis. Transpl..

[111] Baran D.T., Marcy T.W. (1984). Evidence for a defect in vitamin D metabolism in a patient with incomplete Fanconi syndrome. J. Clin. Endocrinol. Metab..

[112] Cunha T.D.S., Heilberg I.P. (2018). Bartter syndrome: Causes, diagnosis, and treatment. Int. J. Nephrol. Renovasc. Dis..

[113] Li X.Y., Jiang Y., Xu L.J., Duan L., Peng X.Y., Chen L.M., Xia W.B., Xing X.P. (2017). A clinical and hereditary analysis of novel complex heterozygous KCNJ1 mutation in a Bartter syndrome type Ⅱ patient. Zhonghua Nei Ke Za Zhi.

[114] Krishnamurthy S., Jagadeesh A. (2017). Bartter Syndrome with Nephrogenic Diabetes Insipidus and Vitamin D Resistant Rickets. Indian Pediatr..

[115] Storm T., Zeitz C., Cases O., Amsellem S., Verroust P.J., Madsen M., Benoist J.F., Passemard S., Lebon S., Jønsson I.M. (2013). Detailed investigations of proximal tubular function in Imerslund-Gräsbeck syndrome. BMC Med. Genet..

[116] Ciancio J.I.R., Furman M., Banka S., Grunewald S. (2019). Profound vitamin D deficiency in four siblings with Imerslund-Grasbeck syndrome with homozygous CUBN mutation. Jimd Rep..

[117] Emma F., Cappa M., Antoniazzi F., Bianchi M.L., Chiodini I., Eller Vainicher C., Di Iorgi N., Maghnie M., Cassio A., Balsamo A. (2019). X-linked hypophosphatemic rickets: An Italian experts’ opinion survey. Ital. J. Pediatr..

[118] Haffner D., Waldegger S., Geary D.F., Schaefer F. (2016). Disorders of phosphorus metabolism. Pediatric Kidney Disease.

[119] Gohil A., Imel E.A. (2019). FGF23 and Associated Disorders of Phosphate Wasting. Pediatr. Endocrinol. Rev..

[120] Conti G., Chirico V., Lacquaniti A., Silipigni L., Fede C., Vitale A., Fede C. (2014). Vitamin D intoxication in two brothers: Be careful with dietary supplements. J. Pediatr. Endocrinol. Metab..

